# The bacterial communities associated with fecal types and body weight of rex rabbits

**DOI:** 10.1038/srep09342

**Published:** 2015-03-20

**Authors:** Bo Zeng, Shushu Han, Ping Wang, Bin Wen, Wensu Jian, Wei Guo, Zhiju Yu, Dan Du, Xiangchao Fu, Fanli Kong, Mingyao Yang, Xiaohui Si, Jiangchao Zhao, Ying Li

**Affiliations:** 1 Farm Animal Genetic Resources Exploration and Innovation Key Laboratory of Sichuan Province, Sichuan Agricultural University, Chengdu, Sichuan, China; 2 Sichuan Academy of Grassland Science, Chengdu, Sichuan, China; 3 Department of Pediatrics and Communicable Disease, University of Michigan, Ann Arbor, Michigan, United States of America

## Abstract

Rex rabbit is an important small herbivore for fur and meat production. However, little is known about the gut microbiota in rex rabbit, especially regarding their relationship with different fecal types and growth of the hosts. We characterized the microbiota of both hard and soft feces from rex rabbits with high and low body weight by using the Illumina MiSeq platform targeting the V4 region of the 16S rDNA. High weight rex rabbits possess distinctive microbiota in hard feces, but not in soft feces, from the low weight group. We detected the overrepresentation of several genera such as *YS2/Cyanobacteria*, and *Bacteroidales* and underrepresentation of genera such as *Anaeroplasma spp.* and *Clostridiaceae* in high weight hard feces. Between fecal types, several bacterial taxa such as *Ruminococcaceae*, *and Akkermansia spp.* were enriched in soft feces. PICRUSt analysis revealed that metabolic pathways such as “stilbenoid, diarylheptanoid, gingerol biosynthesis” were enriched in high weight rabbits, and pathways related to “xenobiotics biodegradation” and “various types of N-glycan biosynthesis” were overrepresented in rabbit soft feces. Our study provides foundation to generate hypothesis aiming to test the roles that different bacterial taxa play in the growth and caecotrophy of rex rabbits.

Rex rabbit is an important small herbivorous mammal widely raised for fur and meat production. Rex rabbit excretes two types of feces: hard feces which contain poorly digestible large particles and soft feces which consist of fermented soft fine particles from caecum fermentation^1^,^2^. As a caecotrophic small animal, rex rabbit eats soft feces, which provides additional proteins, vitamins, and inorganic salt. Earlier studies have shown the differences in nutrients between hard and soft feces^3^,^4^,^5^. However, little is known about the composition of the microbiota of soft and hard feces^6^,^7^.

Gut microbiota play important roles in mammal's health and production. Studies have shown that gut microbiota are associated with many key functions of the host, such as obesity^8^,^9^, gut immune maturation^10^ and nutrition restriction^11^. We thus hypothesize that the gut microbiota differs in fecal types and is also associated with the growth of rex rabbit.

The objectives of this study were: i) to characterize and compare the microbiota in hard and soft feces in rex rabbits and ii) to identify bacterial taxa that are associated with the growth of rex rabbits.

## Methods

### Experimental design and sampling

Our animal experiment was approved by the Institutional Animal Care and Use Committee of the Sichuan Agricultural University under permit number DKY- S20123122 and was performed in the breeding center of rex rabbit research institution located in the suburb of Xinjin County, Chengdu, China. All rex rabbits were fed with customized fodder (probiotics and antibiotics free) and raised under the same temperature (25 ± 1.5°C controlled by automatic heating and ventilation devices). Figure S1 shows the flowchart of this study. Briefly, 80 young female rex rabbits (breed Sichuan White rex rabbit) born on the same day from different rabbit mothers, were raised in separated cages after weaning (day 40) to minimize the confounding effects of genetics and families. Their body weights were sorted on day 70 and the top 10 rex rabbits with the highest weight (HW) and the bottom 10 with the lowest weight (LW) were selected in this study. On day 90, their body weights were measured again and both hard and soft fecal samples were collected.

All experiments were performed in accordance with the approved guidelines and regulations.

For soft fecal sample collection, all rabbits were forced to wear the caecotrophy prevention circle (CP circle, Figure S2) for 10 hours (from 22:00 pm on the day before sampling to 8:00 am on the sampling day) to prevent their caecotrophic behavior. Both the hard and soft feces were automatically dropped into the collection tray under the cage. Fresh fecal samples were immediately transferred into liquid nitrogen container for temporary storage before they were sent to the laboratory where the samples were stored at −80°C.

### DNA extraction and pyrosequencing

Total bacteria DNA was extracted from fecal samples by using PowerFecal™ DNA Isolation kit (MO BIO Laboratories, Carlsbad, CA, USA) according to manufacturer's instruction, and was stored at −80°C before further analysis. Sequencing was performed at the Novogene Bioinformatics Technology Co., Ltd. Briefly, DNA was amplified by using the 515f/806r primer set (515f: 5′-GTG CCA GCM GCC GCG GTA A-3′, 806r: 5′-XXX XXX GGA CTA CHV GGG TWT CTA AT-3′), which targets the V4 region of the bacterial 16S rDNA, with the reverse primer containing a 6-bp error-correcting barcode unique to each sample. PCR reaction was performed using phusion high-fidelity PCR Mastermix (New England Biolabs (Beijing) LTD., China) with the following condition: 94°C for 3 min (1 cycle), 94°C for 45 s/50°C for 60 s/72°C for 90 s (35 cycles), and a last step of 72°C for 10 min. PCR products were purified by using the QIAquick Gel Extraction Kit (QIAGEN, Dusseldorf, Germany). Pyrosequencing was conducted on an Illumina MiSeq 2 × 250 platform according to protocols described by Caporaso, *et al*^12^.

### Bioinformatics and statistical analysis

Sample reads were assembled by using mothur v1.32^13^. Chimeric sequences were removed using the USEARCH software based on the UCHIME algorithm^14^. The microbial diversity was analyzed using the QIIME software^15^ with Python scripts. Operational Taxonomic Unit (OTUs) were picked using *de novo* OTU picking protocol with a 97% similarity threshold. Alpha diversity analysis included Shannon index, Chao1 and observed species. Jackknifed beta diversity included both unweighted and weighted Unifrac distances calculated with 10 times of subsampling, and these distances were visualized by Principal Coordinate Analysis (PCoA)^16^. Taxonomy assignment of OTUs was performed by comparing sequences to the Greengenes database (gg_13_5_otus).

Mann-Whitney U test was used for significance test of alpha diversity. Two-sided Student's t-test was used for significance test of beta diversity difference between sample groups. Linear discriminant analysis coupled with effect size (LEfSe) was performed to identify the bacterial taxa differentially represented between groups at genus or higher taxonomy levels^17^. The functional profiles of microbial communities were predicted by using PICRUSt^18^. Bootstrap Mann-Whitney u-test with 1000 permutations was also used to identify gene pathways or OTUs with significantly different abundance between groups. The R packages “Phyloseq”, “biom”, “pheatmap” were used for data analysis and plotting^19^,^20^.

## Results

### Metadata and sequencing

Not surprisingly, rex rabbit body weights were significantly different between HW and LW groups (Figure S3) on both day 70 and day 90. A total of 40 fecal samples (10 HW hard feces, 10 HW soft feces, 10 LW hard feces and 10 LW soft feces) were collected and sent for sequencing. After OTU picking and chimera checking, a total of 2,078,821 reads were assigned to 60,783 non-singleton OTUs, which resulted in the classification of 474 taxa (genus level). Each sample has 6,904 OTUs and 51,970 sequences on average (Table S1).

### Differences in bacterial communities between high and low weight rex rabbits

**Figure 1:**
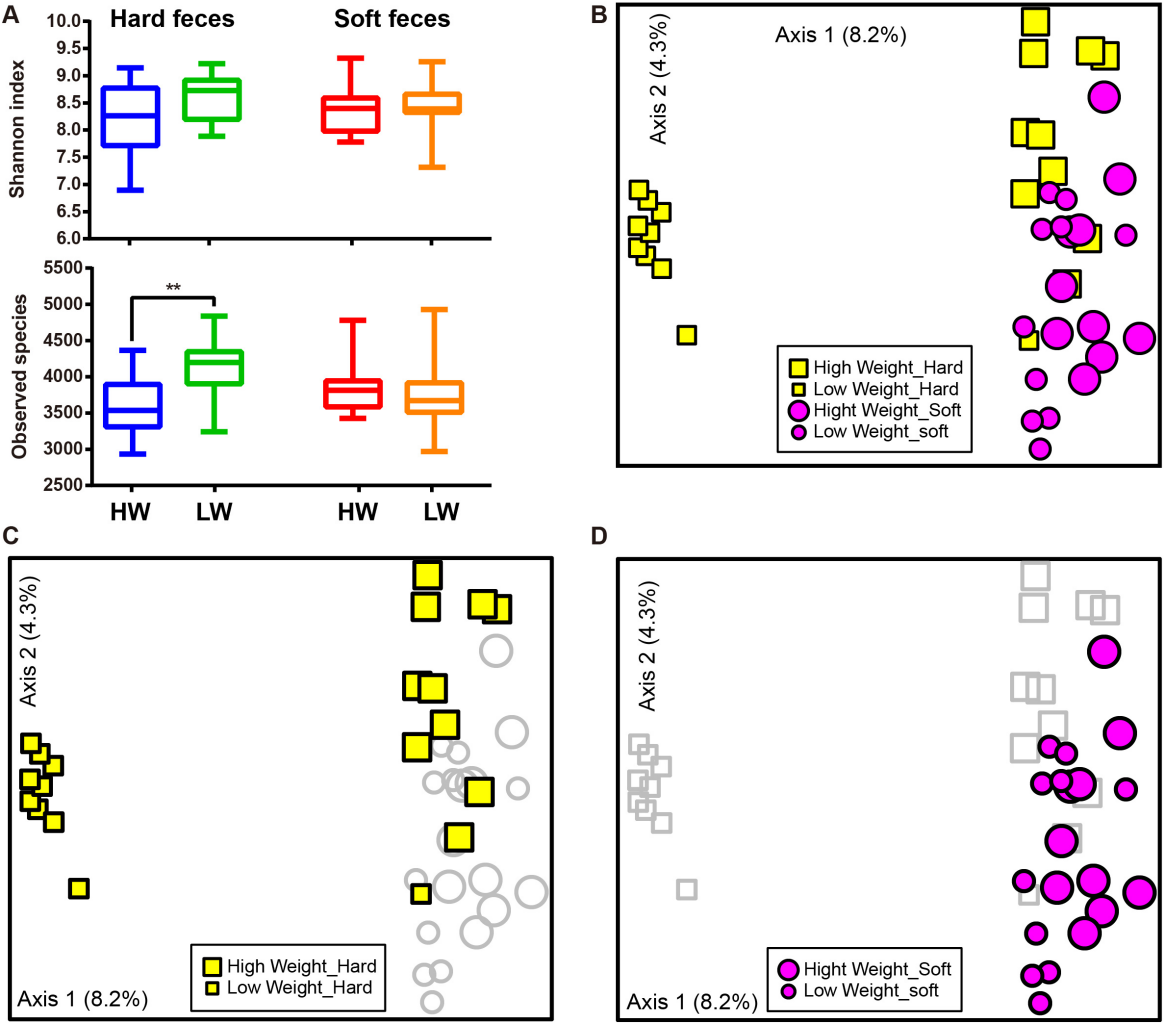
Differences in bacterial community diversity, richness and structures between high and low weight rabbits. (A): Community diversity and richness between high and low weight feces (both hard and soft feces). (B): Principal Coordinate Analysis (PCoA) of bacterial community structures of the gut microbiota of the four sample groups. Each symbol represents each gut microbiota. Bigger symbols represent high weight rabbits. Squares and circles represent hard and soft feces, respectively. (C): PCoA shows distinct bacterial communities between high and low weight hard feces. (D): No significant differences in bacterial communities were observed between high and low Weight soft feces. Sequences were normalized to the depth of 21,522 sequences with 10 times of subsampling to minimize the effect of sequencing depth. Asterisk shows significant differences between groups (** *p* <0.01, * *p* <0.05, Mann-Whitney U test).

Three alpha diversity measures were calculated including Shannon's diversity index, observed species (observed OTUs) (Figure 1A), and Chao1 (estimated OTUs) (Figure S4). We found no significant difference in Shannon diversity between high weight (HW) and low weight (LW) samples (Figure 1A). For community richness comparison, low weight hard feces had significantly higher number of observed and estimated (Chao1) OTUs than high weight hard feces (*p* <0.01, Figure 1A and Figure S4). No significant differences in richness were observed between LW soft and HW soft feces.

The relationships between the community structures of the rex rabbit gut microbiota were examined by using the Principal Coordinate Analysis (PCoA) based on the unweighted and weighted Unifrac distance matrixes. On the PCoA plot, each symbol represents the gut microbiota of a rex rabbit (Figure 1B). Interestingly, the microbiotas of the LW hard feces were distinct from those of the HW hard feces (Figure 1B and C). No significant differences in community structure were observed between the HW and LW soft feces (Figure 1D). The relationships between community structures revealed by PCoA were further tested by comparing the between- and within-group unweighted Unifrac distances. Consistent with the PCoA plot, the between-group distances were significantly higher than the within-group distances (two-tailed Student's t-test, *p* <0.01) for the HW hard and LW hard pair, but not for the HW soft and the LW soft pair (Figure S5). These data suggests that the microbial community structures between HW and LW hard feces were significantly different whereas those between HW vs LW soft were not significantly different (Figure 1 and Figure S5).

**Figure 2:**
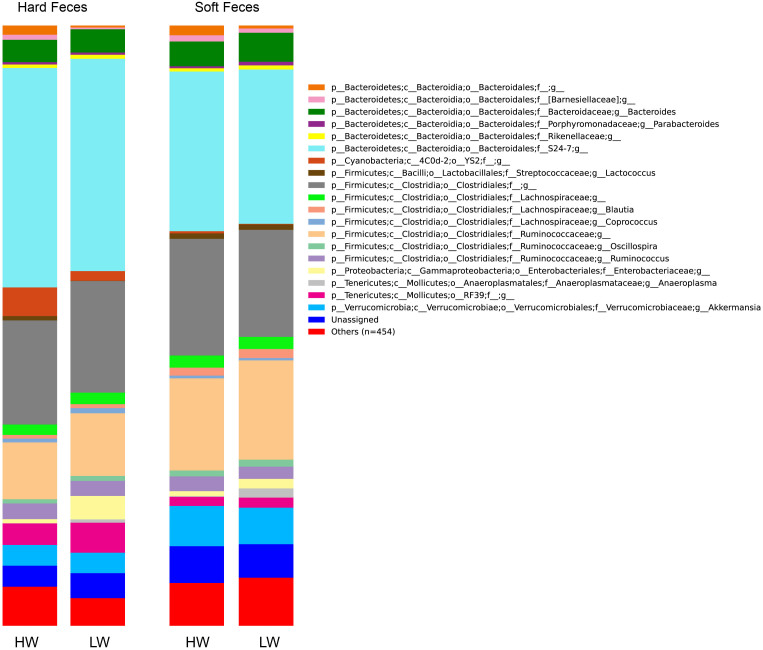
Microbial composition of high and low weight rabbits in different fecal types. Each bar represents the average relative abundance of each bacterial taxon within a group. The top 20 abundant taxa are shown.

Figure 2 shows the community composition of the HW and LW rabbits in the two fecal types. In the stacked bar chart, each bar represents the average relative abundance of each bacterial taxon. The top 20 taxa with high relative abundance, which are in total composed of 93.4% of the reads, were illustrated.

**Figure 3:**
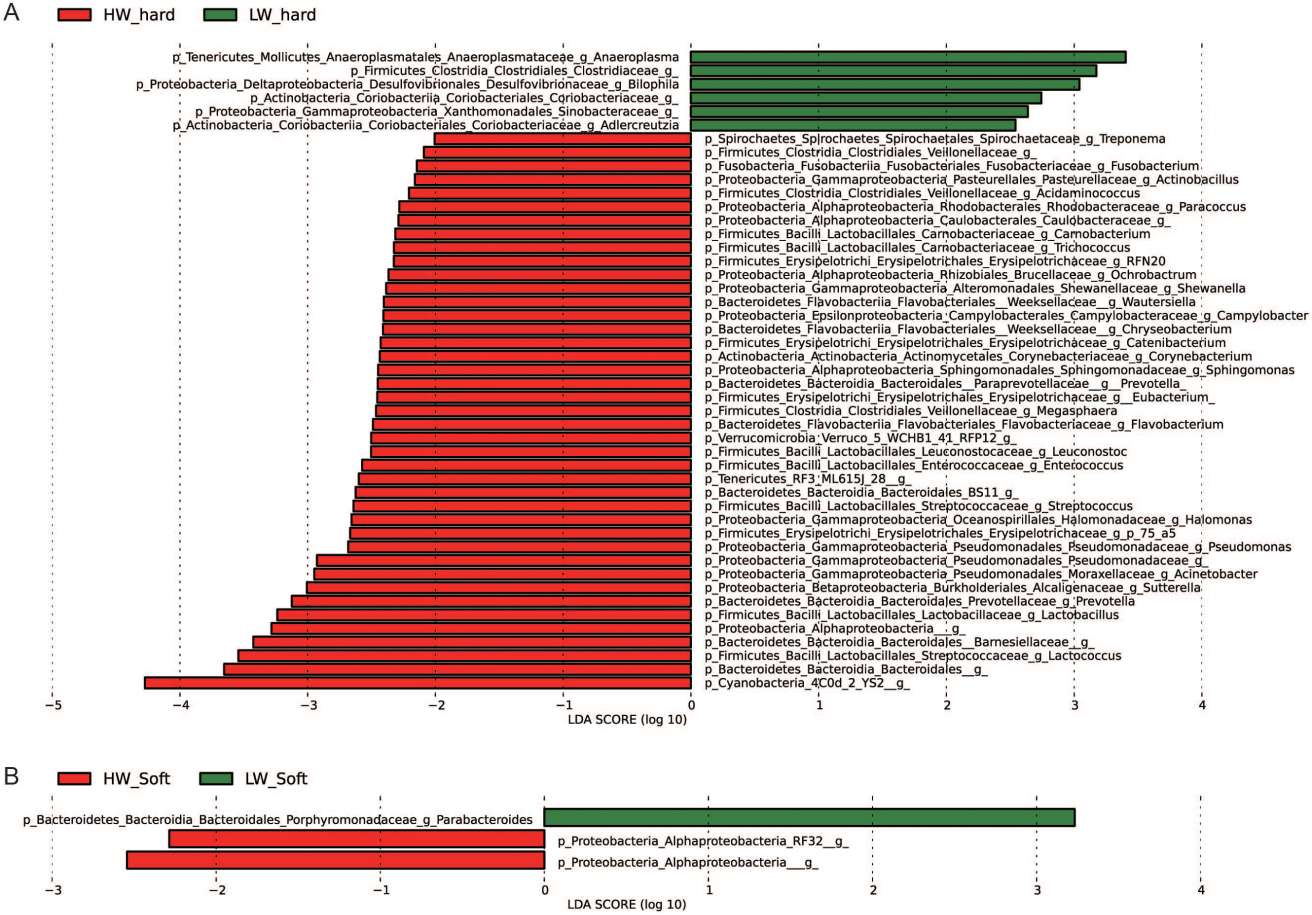
Bacterial taxa significantly differentiated between the high and low weight rabbits identified by linear discriminant analysis coupled with effect size (LEfSe) using the default parameters. (A) and (B) show different taxa between the high weight and low weight rabbits in the hard and soft feces, respectively.

To identify bacterial taxa that were significantly differentiated between groups, we performed LEfSe on 93 top taxa (average relative abundance> 0.0001). This threshold allowed us to keep as many taxa as possible for meaningful comparisons and to eliminate most rare taxa in the analysis. Figure 3 shows bacterial taxa differentially represented between high weight and low weight rabbits. In hard feces, 41 bacterial taxa were significantly more abundant in high weight rabbits (e.g. *YS2*, *Bacteroidales*, *Lactococcus spp.*, *Lactobacillus spp.*, *Prevotella spp.*, *Sutterella spp.*, *Acinetobacter spp*. *p* <0.05), while only 6 taxa were overrepresented in low weight rabbits (e.g. *Anaeroplasma spp.*, *Clostridiaceae*, *p* <0.05) (Figure 3A). As to soft feces, only 3 differentially represented bacterial taxa were detected, with two and one taxa more abundant in high weight and low weight group (*p* <0.05), respectively (Figure 3B).

**Figure 4:**
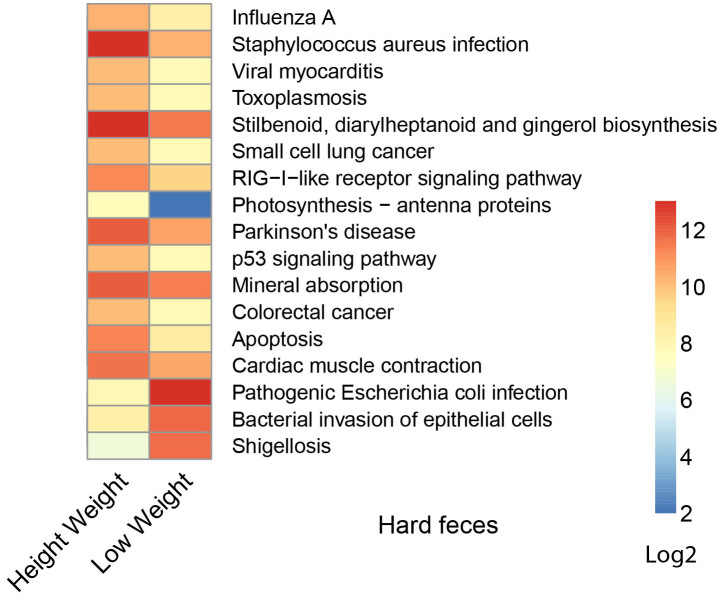
Predicted function of gut micorbiota between the high and low weight hard feces. The gene copy numbers of samples within the same sample group were pooled. Values of each functional gene (row) were log2 transformed. The third level of KEGG pathway was shown in the heatmap. The significant test of the gene distribution between groups were performed using bootstrap Mann-Whitney u-test with cutoffs of *p* <0.01, *FDR* <0.1, Mean counts> 10.

We next used a computational tool, PICRUSt (Phylogenetic Investigation of Communities by Reconstruction of Unobserved States)^20^, to explore the functional profiles of the rex rabbit gut microbiota. The 1123 closed-reference picked and differentially represented OTUs were normalized by 16S rRNA copy number and their metagenomic contributions were predicted from the Kyoto Encyclopedia of Genes and Genomes (KEGG) pathways. In comparison between groups with different weight, 14 pathways (e.g. “Stilbenoid, diarylheptanoid and gingerol biosynthesis”, “Mineral absorption”, “Staphylococcus aureus infection”) were significantly more abundant in the hard feces of high weight rabbits, while other three infectious diseases related pathways (“Pathogenic Escherichia coli infection”, “Shigellosis”, “Bacterial invasion of epithelial cells”) were overrepresented in low weight rabbits (Figure 4, *p*<0.01), and no different pathways were found in their soft feces.

### Differences in bacterial communities between fecal types

**Figure 5:**
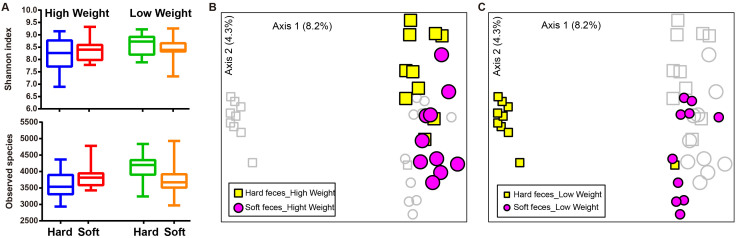
Differences in bacterial community diversity, richness and structures between soft and hard feces. (A): Community diversity and richness between soft and hard feces in high and low weight rabbits. (B): Principal Coordinate Analysis (PCoA) shows distinct bacterial community structures between the hard and soft feces of high weight rabbits. Each symbol represents each gut microbiota. Bigger symbols represent high weight rabbits. Squares and circles represent hard and soft feces, respectively. (C): PCoA shows much more striking differences in bacterial communities between the soft and hard feces in low weight rabbits. Sequences were normalized to the depth of 21,522 sequences with 10 times of subsampling to minimize the effect of sequencing depth.

When comparing alpha diversities between fecal types, no significant differences in diversity or richness were observed between hard and soft feces except a higher Chao1 in hard feces (LW) compared to soft feces (LW) (*p* <0.05, Figure 5A and Figure S3). Whereas PCoA plot based on unweighted Unifrac shows distinct bacterial community structures between hard and soft feces in high weight feces (Figure 5B), much larger and remarkable differences in community structures between fecal types were observed in low weight rabbits (Figure 5C).

**Figure 6:**
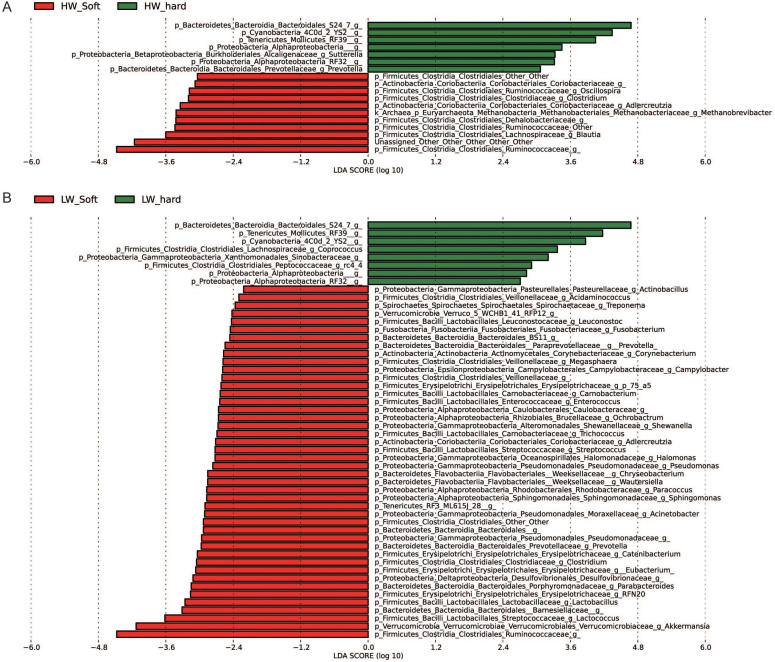
Bacterial taxa significantly differentiated between hard and soft feces identified by linear discriminant analysis coupled with effect size (LEfSe) using the default parameters. (A) and (B) show different taxa between the hard and soft feces in the high weight and low weight rabbits, respectively.

We also performed LEfSe to detect bacterial taxa with significantly different abundance between fecal types (Figure 6). Eleven and forty four taxa were overrepresented in soft feces (e.g. *Ruminococcaceae, Akkermansia spp., Blautia spp., Lactococcus spp., Barnesiellaceae*, *p* <0.05) of high weight rabbits and low weight rabbits, respectively. Seven and eight taxa were identified to be more abundant in hard feces (e.g. *S24-7*, *RF39*, *p <*0.05) of high and low weight groups, respectively.

**Figure 7:**
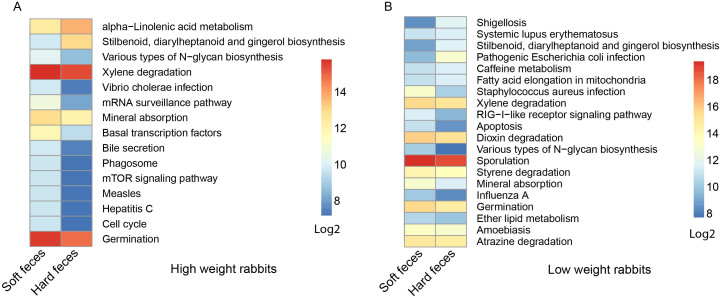
Predicted function of gut micorbiota between the hard and soft feces in high weight (A) and low weight (B) rabbits. The gene copy numbers of samples within the same sample group were pooled. Values of each functional gene (row) were log2 transformed. The third level of KEGG pathway was shown in the heatmap. The significant test of the gene distribution between groups were performed using bootstrap Mann-Whitney u-test with cutoffs of *p* <0.01, *FDR* <0.1, Mean counts> 10.

Similarly, we used PICRUSt to explore the different metabolic potentials between fecal types. A total of 26 pathways (e.g. “Various types of N-glycan biosynthesis”) were more abundant in the soft feces and only 4 pathways were more abundant in hard feces (*p* <0.01). Interestingly, four “xenobiotics biodegradation” pathways (Xylene, Atrazine, Dioxin, Styrene) were enriched in soft feces (Figure 7, bootstrap Mann-Whiteney u-test, *p* <0.01).

## Discussion

### Microbiota related to rabbit body weight

In this study we characterized the gut microbiota in rex rabbits with respect to their correlation with growth and feces types. We found several bacterial taxa such as *YS2/Cyanobacteria*, *Bacteroides*, *Lactococcus spp.,* *Lactobacillus spp.,* and *Prevotella spp.* were overrepresented in high weight hard feces (Figure 3A). *Lactococcus spp.* and *Lactobacillus spp.*, members of which are well-known lactate producing probiotics, are wildly used to improve animal digestion efficiency. *YS2* was previously recognized as a member of *Cyanobacteria*. Recent genomic study showed *YS2* does not have phytosynthetic ability and classified this bacterium into the new class “*Melainabacteria*”. Metabolic analysis demonstrated *YS2* has many special functions including obligate anaerobic fermentation, syntrophic H_2_-production, nitrogen fixation, and synthesis of vitamin B and K^21^.

In human studies, the abundance of *Bacteroides* was reduced in obese children (e.g. Kazakh school children, preschool children of Estonia)^22^,^23^,^24^. However, our study suggests that members of *Bacteroides* were significantly enriched (*p* <0.05) in high weight rabbits. Therefore, not all the members of *Bacteroides* are negatively correlated with body weight. It is also important to note that the high weight rex rabbits are not obese. As small herbivores, rex rabbits contain much lower average fat contents than human. Therefore, members of *Bacteroides* might contribute to the healthy growth of rex rabbits.

Recently, Looft *et al.* (2014) studied the effects of antibiotic on swine gut microbiota and found that Carbadox pre-treatment prevented the increase of *E. coli* populations and promoted a large increase in relative abundance of *Prevotella* populations in these medicated pigs^25^. Consistently, our data also showed increased *Prevotella* and decreased *Enterobacteriaceae* (Figure 3A) in HW rabbits, mirroring the Carbadox mediated growth promotion effect^26^,^27^. Further investigation is warranted to confirm the effect of the differentially represented bacterial taxa in HW and LW groups on the growth of rex rabbits. Based on these studies, it is promising to then develop prebiotics and/or probiotics to promote the “growth positive” and inhibit the “growth-negative” bacterial taxa for the rex rabbits production.

With respect to metabolic pathways, the enrichment of “stilbenoid, diarylheptanoid and gingerol biosynthesis” and “Mineral absorption” pathways in the high weight rex rabbits group are remarkable. Stilbenoid diarylheptanoid and gingerol, known as plant source of phytoalexins were reported to have natural anti-inflammatory or anti-cancer functions^28^,^29^,^30^. Meanwhile, we also found an overrepresentation of “alpha-Linolenic acid metabolism” in hard feces of high weight rabbits (Figure 7A). Alpha-linolenic acid as one of the essential human fatty acids, is often used as nutritional supplement. It can be converted into Eicosapntemacnioc acid (EPA), Docosahexenoic acid (DHA) *etc.* in the body, and lead to many health related functions like anti-inflammatory effects, or prevention of stroke and heart disease^31^,^32^,^33^. The enrichment of these health-related pathways might contribute to the higher weight of rex rabbits. Besides, the high abundance of infectious diseases pathways in low weight rabbits was construable due to its high *Enterobacteriaceae* content. However, none of the rabbits had symptoms associated with bacterial infection. Therefore, either these pathways were not expressed, or did not reach a level to cause the onset of symptoms.

Further experiments such as transcriptomics, metabolomics and fecal transplant are needed to verify the functions and roles that these high-weight enriched bacteria play in rabbit growth.

### Specific gut bacteria in rabbit soft feces

Although similar microbial richness was observed between hard and soft feces, the community structures are significantly different between fecal types. Both in the HW and LW rabbit group, the soft fecal microbiota is strikingly separated from the hard fecal microbiota. Members of the *Akkermansia spp.*, *Blautia spp.*, and *Oscillospira spp.*, are three of the several bacterial genera that were overrepresented in the soft fecal microbiota.

*Akkermansia spp.* has been detected in the intestines of many vertebrates such as human^34^, mouse^35^, and zebrafish^36^. Recent studies suggested *Akkermansia spp*. plays an important role in mucus degradation and production^37^. The enrichment of *Akkermansia* in rabbit soft feces is in keeping with the hypothesis that members of this bacterial taxon might contribute to the formation of the mucus-covered soft feces.

*Blautia sp.* has been proved to be one of the core microflora of many mammals. Although the relative abundance of *Blautia spp.* negatively correlated with many diseases such as *Clostridium difficile* infection^38^, type 1 diabetes^39^, acute hemorrhagic diarrhea^40^, cirrhosis^41^, some species of this genus such as *B. faecis* and *B.* *stercoris* can utilize carbohydrates as fermentable substrates, and produce acetate and lactate as the major end products of glucose fermentation^42^,^43^. In addition, Godwin (2013) reported the *B. coccoides* as a reductive acetogen which are associated with carbon dioxide and hydrogen metabolism and results in reduced methane output of kangaroos^44^. Consistent with these studies, the enrichment of *Blautia* spp. in rabbit soft feces indicates members of this genus might play roles in the digestion of the diets in cecum.

*Oscillospira spp.* has been detected in the rumen of several herbivores such as cattle, sheep and reindeer^45^. Recently, members of this bacterium have been shown to be prevalent in macropod^46^ and humans^47^ as well. The overrepresentation of this genus in rabbit soft feces indicates that members of this genus might be involved in fermentation, as soft feces are the product of the caecum fermentation.

Several metabolic pathways were enriched in soft feces including bile secretion, mineral absorption, and xenobiotics biodegradation. Bile secretion and mineral absorption belong to the digestive system pathway, although their relationship with gut bacteria is still unclear. We found four pathways related to xenobiotics biodegradation are enriched in soft feces, including atrazine, dioxin, styrene, and xylene degradation. Atrazine is a broad-spectrum herbicide. Dioxin is also wildly contained in many pesticides. These compounds are considered toxic, mutagenic, and possibly carcinogenic. The enrichment of these pathways in soft feces suggests that the gut bacteria in rabbit cecum may also have detoxification function.

The enrichment of “Various types of N-glycan biosynthesis” pathway in soft feces may be due to the presence of higher abundant *Campylobacter spp.* in soft feces (Figure 6B). This pathway, first discovered in *Campylobacter jejuni*^48^, directly related to N-linked protein glycosylation which influences multiple protein functions including sorting, targeting, localization, stability, and quality control of protein synthesis. These functions were proved to enhance bacterial fitness by protecting bacterial proteins from cleavage by the gut proteases^49^.

It is worth noting that the accuracy of PICRUSt in predicting bacterial metabolic pathways depends much on the available reference bacterial genomes in the database. Although high agreement has been reached between PICRUSt predictions and human metagenome data across all body sites, the relevance of application of PICRUSt to predict bacterial activities in rabbits gut needs further validation. The average Nearest Sequenced Taxon Index (NSTI, 0.195 ± 0.05 s.d.) of our samples is comparable to those of other mammals (0.14 ± 0.06) and soils (0.17 ± 0.02). Despite the accurate metagenome predictions for soil samples and a subset of mammals (NSTI<0.05), the prediction accuracy of PICRUSt in rabbits needs further validation. Furthermore, a large portion of the OTUs were not matched to the database, thus their functions were not imputed. Of note, some human related pathways were identified probably due to poor annotation in the KEGG database and/or homologous pathways between bacteria and humans. These pathways were not discussed in this study. Nevertheless, despite these potential biases, PICRUSt provide important insight into bacterial community functions in rabbit gut. Other omics approaches (e.g. transcriptomics and metabolomics) are desired to confirm these discoveries and improve our understanding of the bacterial functions in rabbit guts.

In summary, we characterized the gut microbiota in soft and hard feces of rex rabbits with different weight. We identified bacterial taxa and metabolomic pathways that were overrepresented in different fecal groups. These features serve as immediate targets for future studies to test their roles in the growth and caecotrophic behavior of rex rabbits.

## Author Contributions

Conceived and designed the experiments: B.Z., P.W., S.H., J.Z. and Y.L. Performed the experiments: B.Z., P.W. and S.H. Contributed reagents/materials/analysis tools: B.W., W.J., Z.Y., D.D., W.G., X.F., F.K., X.S. and M.Y. Wrote the paper: B.Z., J.Z. and Y.L.

## Supplementary Material

Supplementary Information Supplementary Information
