# Supplementary material for: The bacterial communities associated with fecal types and body weight of rex rabbits
Source: Sci Rep. 2015 Mar 20;5:9342. doi: 10.1038/srep09342 (PMC4366860; doi:10.1038/srep09342)
Supplement: Supplementary Information [file srep09342-s1.pdf]

The bacterial communities associated with fecal types and body weight of rex rabbits

Bo Zeng[1#], Ping Wang[2#], Shushu Han[1#], Bin Wen[2], Wensu Jian[2], Wei Guo[1], Zhiju Yu[2], Dan Du[2], Xiangchao Fu[2], Fanli Kong[1], Xiaohui Si[1], Mingyao Yang[1], Jiangchao Zhao[3*] and Ying Li[1*]

[1]Farm Animal Genetic Resources Exploration and Innovation Key Laboratory of Sichuan Province, Sichuan Agricultural University, Chengdu, Sichuan, China.

[2]Sichuan Academy of Grassland Science, Chengdu, Sichuan, China.

[3]Department of Pediatrics and Communicable Disease, University of Michigan, Ann Arbor, Michigan, United States of America.

Table S1 OTU table summary

Rabbit ID	Sample Type	Weight group	Group	Sample ID	Sequence counts	Observations (OTU number)
rex1	hard	HW	HW_hard	1	56878	6926
rex5	hard	HW	HW_hard	2	55695	6546
rex3	hard	HW	HW_hard	3	23511	3912
rex2	hard	HW	HW_hard	4	33591	4504
rex6	hard	HW	HW_hard	5	62241	8046
rex10	hard	HW	HW_hard	6	74060	6930
rex4	hard	HW	HW_hard	7	54363	5517
rex8	hard	HW	HW_hard	8	58505	7604
rex18	hard	HW	HW_hard	9	77263	10103
rex9	hard	HW	HW_hard	10	49087	5745
rex28	hard	LW	LW_hard	11	56015	8075
rex37	hard	LW	LW_hard	12	34108	6599
rex35	hard	LW	LW_hard	13	48055	7338
rex36	hard	LW	LW_hard	14	51203	7835
rex24	hard	LW	LW_hard	15	48992	6895
rex31	hard	LW	LW_hard	16	35424	5278
rex25	hard	LW	LW_hard	17	43715	6839
rex38	hard	LW	LW_hard	18	57028	8738
rex32	hard	LW	LW_hard	19	44097	7094
rex40	hard	LW	LW_hard	20	46874	5586
rex1	soft	HW	HW_soft	21	53227	6919
rex5	soft	HW	HW_soft	22	76097	9280
rex3	soft	HW	HW_soft	23	56971	6908
rex2	soft	HW	HW_soft	24	58363	7710
rex6	soft	HW	HW_soft	25	60419	9490
rex10	soft	HW	HW_soft	26	47759	6102
rex4	soft	HW	HW_soft	27	33243	4634
rex8	soft	HW	HW_soft	28	44660	6399
rex18	soft	HW	HW_soft	29	61449	7871
rex9	soft	HW	HW_soft	30	66317	7966
rex28	soft	LW	LW_soft	31	48294	6196
rex37	soft	LW	LW_soft	32	44525	6201
rex35	soft	LW	LW_soft	33	55451	7864

rex36	soft	LW	LW_soft	34	30663	4864
rex24	soft	LW	LW_soft	35	50634	6907
rex31	soft	LW	LW_soft	36	57090	5859
rex25	soft	LW	LW_soft	37	60926	6922
rex38	soft	LW	LW_soft	38	54996	9074
rex32	soft	LW	LW_soft	39	64424	7359
rex40	soft	LW	LW_soft	40	42608	5561

Figure S1

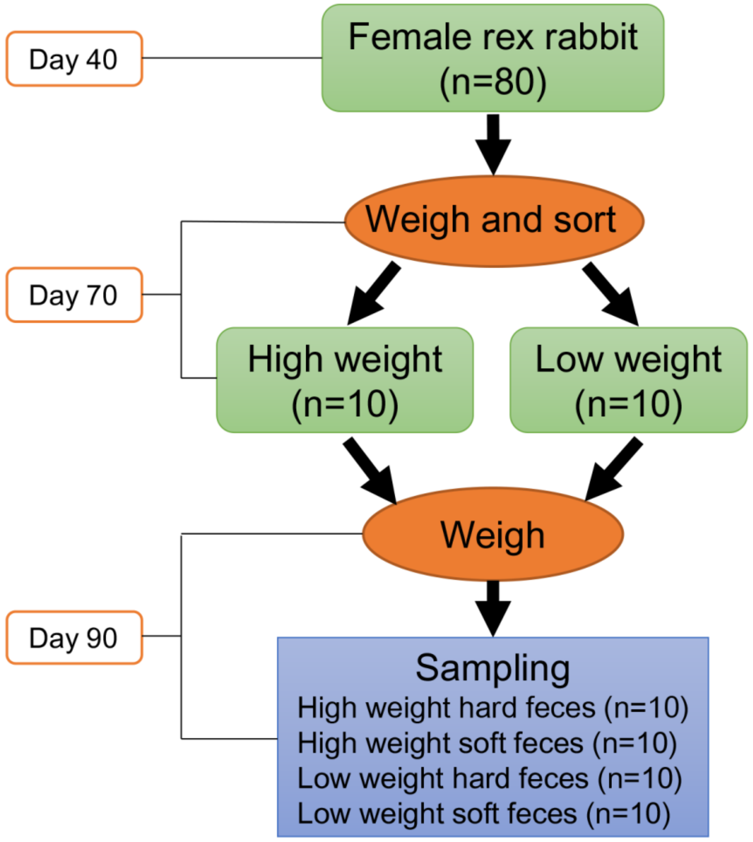

Figure S1. Flowchart of the experiment including rabbit grouping and sampling.

Figure S2

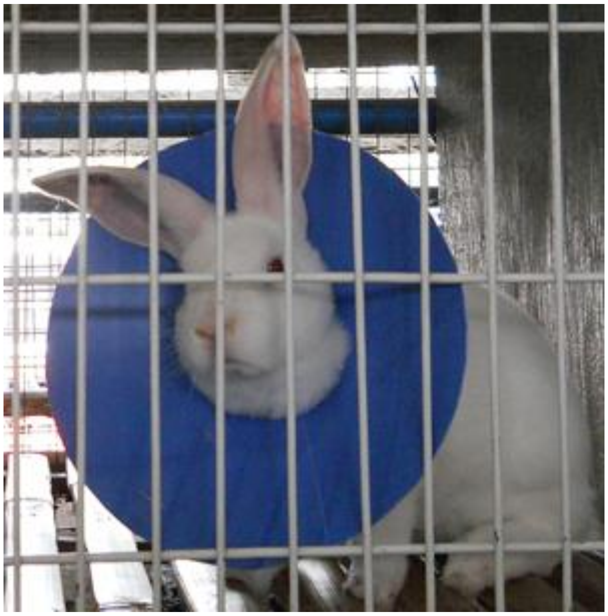

Figure S2. Caecotrophy prevention (CP) circle used to prevent the rabbits from eating their soft feces during sampling. The CP circle is a blue, plate shaped neck collar made with 1 mm sick PP plastic. With its moderate rigidity and angularity, it can only prevent the rabbit from eating their own soft feces, without disturbing normal food ingestion, drinking, moving or sleeping in cages. Photo was taken by B.Z.

Figure S3

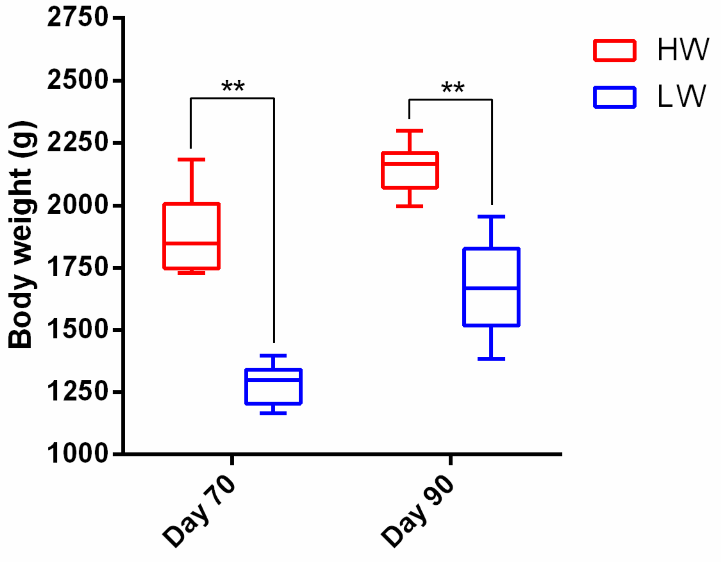

Figure S3. Comparison of body weight between rabbit groups. HW: High weight group (n=10). LW: Low weight group (n=10). Asterisk represents the significance test (***p* < 0.01, One-sided Student's t-test).

Figure S4

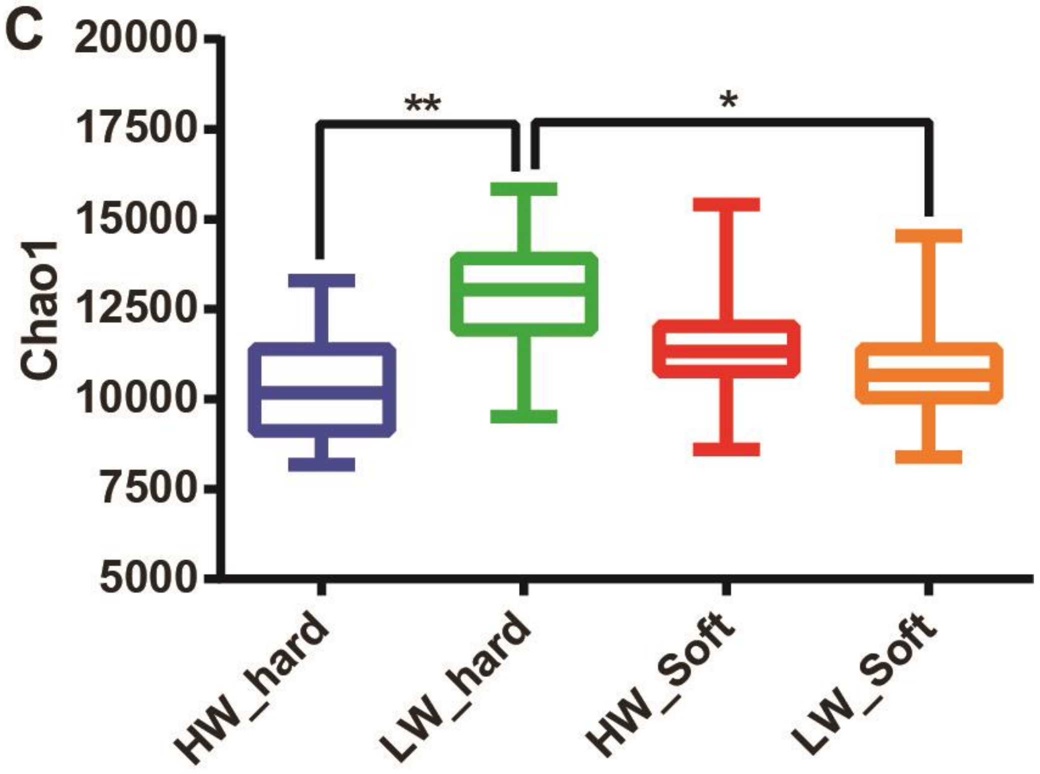

Figure S4. Differences in bacterial community richness (Chao1) between the four groups. Asterisk shows significant differences between groups (** $p < 0.01$, * $p < 0.05$, Mann-Whitney U test).

Figure S5

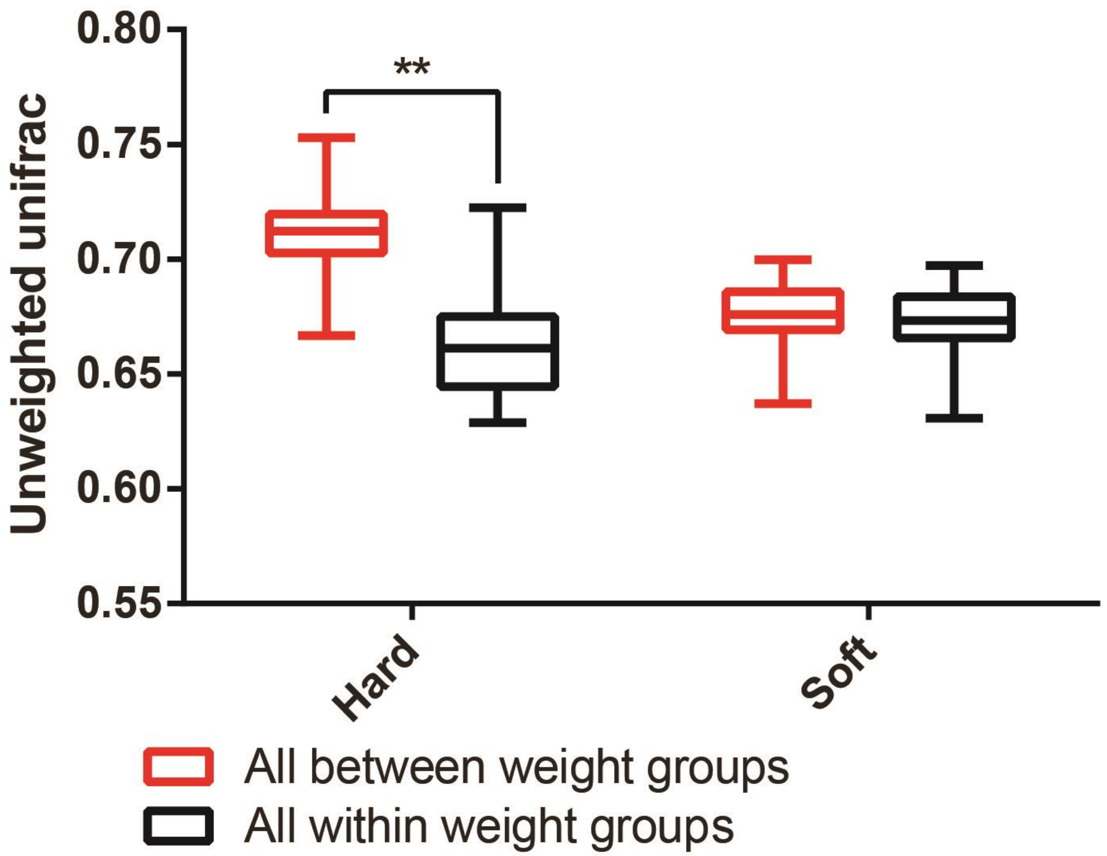

Figure S5. Comparison of the unweighted Unifrac distances between high and low weight groups in hard and soft feces (** $p < 0.01$, two-sided Student's two-sample t-test).
